# Harnessing the Electronic Health Record and Computerized Provider Order Entry Data for Resource Management During the COVID-19 Pandemic: Development of a Decision Tree

**DOI:** 10.2196/32303

**Published:** 2021-10-18

**Authors:** Hung S Luu, Laura M Filkins, Jason Y Park, Dinesh Rakheja, Jefferson Tweed, Christopher Menzies, Vincent J Wang, Vineeta Mittal, Christoph U Lehmann, Michael E Sebert

**Affiliations:** 1 Department of Pathology University of Texas Southwestern Medical Center Dallas, TX United States; 2 Department of Advanced Analytics and Informatics Children's Health Dallas, TX United States; 3 Division of Pediatric Emergency Medicine Department of Pediatrics University of Texas Southwestern Medical Center Dallas, TX United States; 4 Division of Pediatric Hospital Medicine Department of Pediatrics University of Texas Southwestern Medical Center Dallas, TX United States; 5 Clinical Informatics Center University of Texas Southwestern Medical Center Dallas, TX United States; 6 Department of Pediatrics University of Texas Southwestern Medical Center Dallas, TX United States; 7 Department of Population and Data Sciences University of Texas Southwestern Medical Center Dallas, TX United States; 8 Lyda Hill Department of Bioinformatics University of Texas Southwestern Medical Center Dallas, TX United States; 9 Division of Infectious Diseases Department of Pediatrics University of Texas Southwestern Medical Center Dallas, TX United States

**Keywords:** COVID-19, computerized provider order entry, electronic health record, resource utilization, personal protective equipment, SARS-CoV-2 testing, clinical decision support

## Abstract

**Background:**

The COVID-19 pandemic has resulted in shortages of diagnostic tests, personal protective equipment, hospital beds, and other critical resources.

**Objective:**

We sought to improve the management of scarce resources by leveraging electronic health record (EHR) functionality, computerized provider order entry, clinical decision support (CDS), and data analytics.

**Methods:**

Due to the complex eligibility criteria for COVID-19 tests and the EHR implementation–related challenges of ordering these tests, care providers have faced obstacles in selecting the appropriate test modality. As test choice is dependent upon specific patient criteria, we built a decision tree within the EHR to automate the test selection process by using a branching series of questions that linked clinical criteria to the appropriate SARS-CoV-2 test and triggered an EHR flag for patients who met our institutional persons under investigation criteria.

**Results:**

The percentage of tests that had to be canceled and reordered due to errors in selecting the correct testing modality was 3.8% (23/608) before CDS implementation and 1% (262/26,643) after CDS implementation (*P*<.001). Patients for whom multiple tests were ordered during a 24-hour period accounted for 0.8% (5/608) and 0.3% (76/26,643) of pre- and post-CDS implementation orders, respectively (*P*=.03). Nasopharyngeal molecular assay results were positive in 3.4% (826/24,170) of patients who were classified as asymptomatic and 10.9% (1421/13,074) of symptomatic patients (*P*<.001). Positive tests were more frequent among asymptomatic patients with a history of exposure to COVID-19 (36/283, 12.7%) than among asymptomatic patients without such a history (790/23,887, 3.3%; *P*<.001).

**Conclusions:**

The leveraging of EHRs and our CDS algorithm resulted in a decreased incidence of order entry errors and the appropriate flagging of persons under investigation. These interventions optimized reagent and personal protective equipment usage. Data regarding symptoms and COVID-19 exposure status that were collected by using the decision tree correlated with the likelihood of positive test results, suggesting that clinicians appropriately used the questions in the decision tree algorithm.

## Introduction

COVID-19 is caused by SARS-CoV-2 and has quickly emerged as a global pandemic since its initial description in December 2019 [1]. The increased testing and isolation of patients with COVID-19 are important means of limiting the spread of infection. Many laboratories in the United States have expanded their testing capabilities rapidly [2]. As a result, the overall testing capacity in the United States is substantially larger than what the Centers for Disease Control and Prevention and state health agencies were able provide at the start of the pandemic. Testing shortages however persisted throughout 2020 and, to a lesser extent, into 2021 due to inadequate supplies of collection swabs, viral transport media, RNA extraction regents, and other reagents and consumables [3,4]. Institutions have had to prioritize testing by taking into account the severity of illnesses, the rapidness of results, bed availability, and staffing needs [4].

Electronic health records (EHRs) and computerized provider order entry (CPOE) systems offer the potential to reduce the number of medical errors and improve care quality by facilitating communication, providing access to information, monitoring patients, providing decision support, and enhancing clinicians’ situational awareness [5-7]. However, EHRs can also inadvertently result in clinicians introducing new errors, overlooking existing orders, and duplicating work [8-10]. Apart from the need to reduce costs, preventing the duplicate testing of patients for COVID-19 is essential for conserving existing testing supplies and maximizing the number of patients that can be tested.

Although the availability of testing is important, so is the timely dissemination of test results to care providers to optimally allocate valuable hospital resources, such as limited supplies of personal protective equipment (PPE), effectively [4]. Testing capacities have increased since the early days of the pandemic, but the proliferation of different testing platforms and methodologies has led to variations in test turnaround times and assay sensitivity. Commercial vendors have produced high-throughput, cartridge-based instruments that promise shorter testing turnaround times; however, the demand for these instruments currently exceeds the amount of available supplies [4].

To meet the testing needs of our patient population despite equipment shortages, institutions such as our pediatric health care system had to assemble a variety of COVID-19 testing modalities with varying performance characteristics. Matching testing modalities to the appropriate clinical scenario was a challenge. Some institutions developed decision-making algorithms to stratify their patient population into risk groupings [11]. Herein, we describe and evaluate the CPOE clinical decision support (CDS) tools that were developed to optimize the ordering of COVID-19 tests; the EHR functionalities that were leveraged to manage persons under investigation (PUIs); and the data analysis tools that were essential for monitoring changing variables, such as ordering patterns and available reagent supplies.

## Methods

### Setting and Institutional Approach to Managing the COVID-19 Pandemic

Our academically affiliated pediatric health care system in North Texas consists of 3 acute care hospitals that are licensed for a total of 601 beds and 24 ambulatory specialty care centers. Together, these facilities care for more than 227,000 unique patients per year and have provided services, including more than 19,600 surgeries and 107,800 emergency department visits [12]. Our health system’s efforts in preparing for patients with COVID-19 began early in 2020 and included the activation of the Hospital Incident Command Structure on March 5. A sick isolation unit was opened on March 23 for the management of patients who did not require critical care and were either suspected of SARS-CoV-2 infection—designated as PUIs—or confirmed to be infected. The first positive SARS-CoV-2 test result for a patient in our system was received later that month (March 31). With the activation of the Hospital Incident Command Structure, we recognized that the pandemic would require an organized, sustainable, and adaptable approach to caring for children with COVID-19 while minimizing staff exposure and optimizing the use of PPE and testing reagents and supplies. In this study, we describe and evaluate tools that were developed within the EHR and were vital components of this approach.

As COVID-19 spread across the world and within the United States, the epidemiology of the disease morphed over time. First, cases were seen predominately among patients who had been exposed to the disease during recent travel. Afterward, the disease began to spread within communities, but most new infections were still identified among individuals who had contact with a limited number of confirmed local cases. Finally, widespread community transmission developed, and many cases could no longer be reliably related to a known exposure or travel history [13-15]. In early 2020, the criteria recommended by the Centers for Disease Control and Prevention for identifying a person as a PUI changed several times [16,17]. Reflecting the changing disease epidemiology, these PUI definitions, which had initially focused on symptomatic individuals with a history of travel to Wuhan, China, or a history of contact with a laboratory-confirmed case of COVID-19, were later expanded by the addition of criteria related to travel from mainland China, travel from affected geographic areas within the United States, and, finally, even individuals with no known exposure risk factors [16]. Following the initial pandemic period, during which SARS-CoV-2 testing was available at our institution only through public health laboratories, the options for testing increased first thanks to offerings from commercial reference laboratories and then due to the launch of an internal, laboratory-developed test with a turnaround time of approximately 24 hours. Later, our laboratory implemented commercial rapid testing platforms that offered further improvements in turnaround times for a limited number of specimens depending on the availability of the required kits (Table 1).

**Table 1:** The SARS-CoV-2 assays implemented.

Assay characteristic	Modified CDC^a^ SARS-CoV-2 Assay (laboratory-developed test)	Biofire Respiratory Panel 2.1 (bioMérieux SA)	Xpert Xpress SARS-CoV-2 (Cepheid)	SARS^b^ Antigen FIA^c^ (Quidel Corporation)	Alinity m SARS-COV-2 Assay (Abbott Laboratories)^d^	Cobas SARS-CoV-2 (Roche Holding AG)^e^
Analyte	RNA	RNA	RNA	Antigen	RNA	RNA
Sample collection	NP^f^ swab in UTM^g^	NP swab in UTM	NP swab in UTM	Anterior nares swab	NP swab in UTM	NP swab in UTM
SARS-CoV-2 target	Nucleocapsid gene	Membrane gene and surface gene	Envelope gene and nucleocapsid 2 gene	Nucleocapsid protein	Nucleocapsid gene and RdRp^h^ gene	Envelope gene and RdRp gene
SARS-CoV-2 LoD^i^	260 copies/mL	160 copies/mL	250 copies/mL	113 TCID50^j^/mL	100 copies/mL	0.003 TCID50/mL
Other target(s)	None	21 additional viruses and bacteria	None	None	None	None
Instrument(s)	EMAG (extraction; bioMérieux SA) and ABI^k^ 7500 (polymerase chain reaction; Thermo Fisher Scientific)	FilmArray Torch System (bioMérieux SA)	GeneXpert XVI (Cepheid)	Sofia 2 (Quidel Corporation)	Alinity m System (Abbott Laboratories)	Cobas 6800 (Roche Holding AG)
Maximum throughput^l^	150 samples/8-hour shift (extraction and polymerase chain reaction)	<1 hour/test/ instrument module	<1 hour/test/ instrument module	20 min/test/ instrument module	300 tests/8-hour shift	864 tests/8-hour shift
Time to results, mean (SD)^m^	0.79 (0.85) days	70 (17) min	77 (29) min	27 (5) min	0.53 (0.35) days	2.03 (1.56) days

^a^CDC: Centers for Disease Control and Prevention.

^b^SARS: severe acute respiratory syndrome.

^c^FIA: fluorescent immunoassay.

^d^The assay was performed at reference lab 1.

^e^The assay was performed at reference lab 2.

^f^NP: nasopharyngeal.

^g^UTM: universal transport medium.

^h^RdRp: RNA-dependent RNA polymerase.

^i^LoD: limit of detection (the LoD shown is either the lowest reported [highest sensitivity] value on the package insert or the lowest value observed in the laboratory).

^j^TCID50: median tissue culture infectious dose.

^k^ABI: Applied Biosystems.

^l^Maximum throughput assumes sufficient reagents. Maximum throughput volumes were not achieved for most platforms due to limited reagent allocations.

^m^The time from specimen (primary orders) or order (add-on orders) receipt in the lab to result reporting. This includes transport to outside labs (send-out testing only), laboratory processing, sample preparation, instrument time, and result reporting.

New institutional policies and procedures, in response to the COVID-19 pandemic, were instituted in parallel with the changed understanding of the disease’s epidemiology, the illness, and SARS-CoV-2 transmission. These changes included the adoption (on April 28, 2020) of universal SARS-CoV-2 testing for all patients who were admitted through the emergency department or directly to inpatient floors and the intensive care unit. At first, rapid testing was prioritized for patients with fevers or respiratory symptoms or those who had close contact with individuals with SARS-CoV-2 infection, while other patients were tested by using the laboratory-developed test. This strategy directed limited resources for rapid testing toward patients with the highest likelihood of infection but resulted in a delay in identifying asymptomatic positive cases, which represent a considerable portion of SARS-CoV-2 infections in children. As rapid testing became increasingly available, such tests were deployed subsequently for all admitted patients.

To optimize the use of resources, such as negative pressure rooms and PPE, we developed a policy for aerosol-generating procedures (AGPs). The policy governed the performance of AGPs, including any preceding SARS-CoV-2 testing and PPE requirements, in a systematic manner that was driven by patients’ symptoms, their COVID-19 status (if known), the prevalence of infection in the community, and the classification of AGPs into 2 risk tiers. SARS-CoV-2 testing was initially required in advance for all patients undergoing scheduled surgery, and the empiric use of PPE, including N95 respirators, was reserved for urgent or emergent cases when testing was not feasible. As community spread increased and access to rapid testing improved, the testing requirement was extended to any urgent surgical procedures for which sufficient time was available.

### EHR Decision Tree for SARS-CoV-2 Test Order Placement

**Figure 1:**
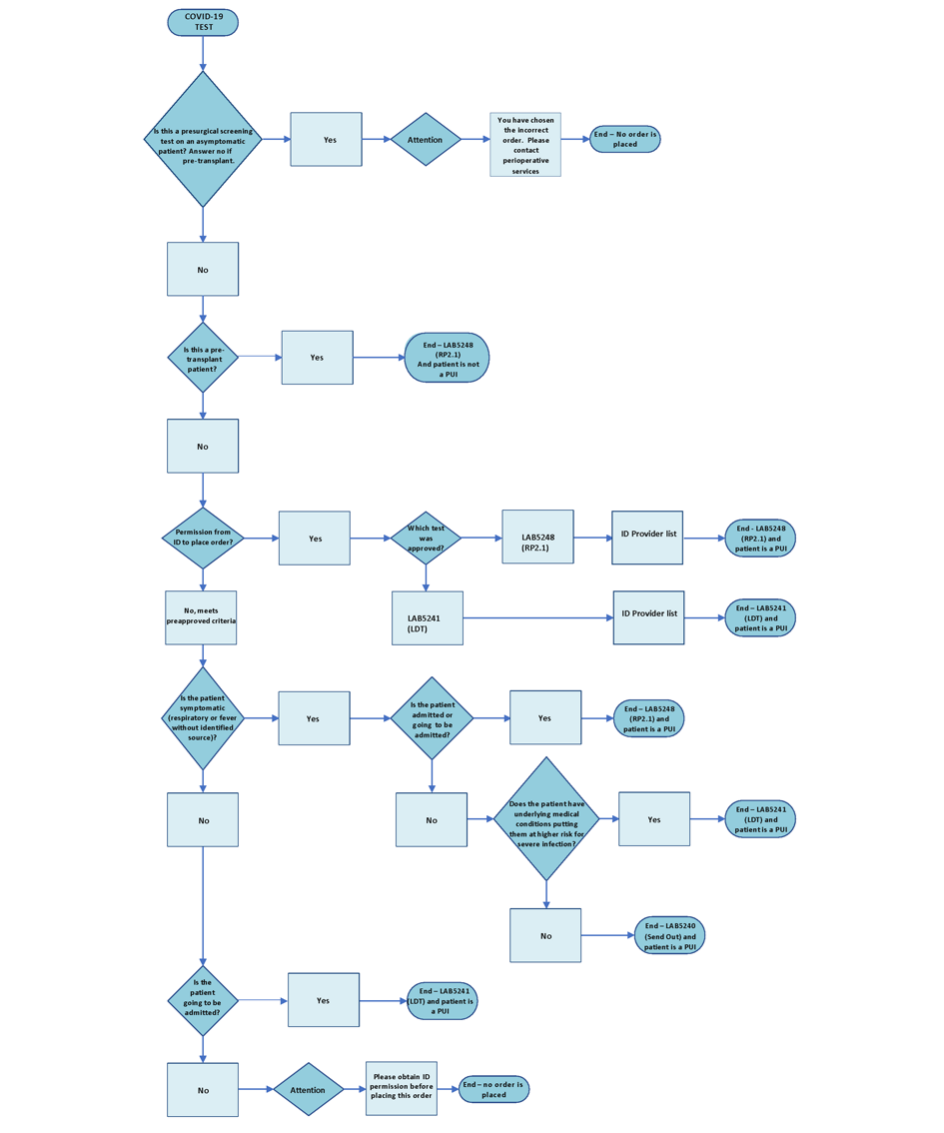
Electronic health record decision tree for ordering SARS-CoV-2 tests. This flow diagram shows the branching set of hierarchical questions that resulted in the capture of data for test prioritization and symptom status identification. LDT: laboratory-developed test; PUI: person of interest; RP2.1: BioFire Respiratory Panel 2.1.

Given the scarcity of testing resources and growing demand during the early phase of the pandemic, formal criteria for SARS-CoV-2 testing were developed at our institution through consensus among physician and clinical laboratory leaders. Prior to the pandemic, our institution did not restrict the ordering of assays for non–SARS-CoV-2 respiratory viruses nor collect data on the reasons for ordering such tests systematically. Developing an ordering system that would be intuitive for clinicians to use and would capture data to guide the prioritization of orders and subsequent revisions to indications for ordering were therefore important priorities. However, the criteria for ordering specific COVID-19 tests were complex, and the metadata were frequently revised as new clinical scenarios were incorporated and new testing options became available. The implementation of the detailed ordering criteria in the EHR posed a challenge that increased with the number of available testing options. More importantly, the growing list of testing indications was a hard-to-navigate obstacle for care providers who needed to place orders. To ease the burden of ordering the correct test from a long list of choices, we built a decision tree within the EHR to automate the selection process based on answers that are provided to a branching set of hierarchical questions. This decision tree (Figure 1) was first implemented on April 28, 2020, and was subsequently updated and frequently modified during the early response to the pandemic.

### PUI Flagging in the EHR

In addition to linking clinical indications to the appropriate SARS-CoV-2 test, the ordering process required setting a flag in the EHR for any patient who met our institutional PUI criteria. The flag alerted health care personnel to a patient’s PUI status and the need to use PPE beyond those for standard precautions, including N95 respirators or powered air purifying respirators, when caring for patients.

Testing for an infectious disease usually suggests a clinical index of suspicion that, in itself, may justify flagging patients in the EHR for the possibility of being infected with that disease. In the case of COVID-19 however, institutional policies required SARS-CoV-2 testing upon admission or before surgery for all patients, even in the absence of symptoms or exposure, thereby rendering the presence of an ordered test functionally meaningless as an indicator of clinical suspicion. Although some patients may be asymptomatic carriers and thus could expose the workforce, the pretest probability of infection in such patients was not expected to be above that of the general population. The universal usage of N95 respirators for all health care encounters during the pandemic was neither recommended or feasible, given the limited supplies. Therefore, our institution decided that patients without compatible symptoms or recent exposure to SARS-CoV-2 would not be designated as PUIs, even when routine testing is required by institutional screening protocols. Consequently, in addition to guiding the selection of the correct SARS-CoV-2 test, the decision tree needed to assign the appropriate PUI status to each patient based on the indication for testing.

The introduction of additional testing modalities with decreased sensitivity compared to that of molecular nasopharyngeal sample testing modalities presented another challenge. Although positive results from these less sensitive assays were considered reliable, negative results were not and required confirmation with a more sensitive molecular test. Accordingly, the EHR rules for the clearance of PUI flags were constructed to require a negative molecular test from a nasopharyngeal sample, even if the flag had originally been triggered by an order for a less sensitive screening test.

A flagging system was also created for displaying results from SARS-CoV-2 tests that had been performed at outside facilities with interoperable EHRs. Such outside test results were either flagged as being reliable and approved by our system’s laboratory as being equivalent to internal testing results (by the Happy Together EHR collaborative, which includes Children’s Health, Parkland Hospital, and University of Texas Southwestern Medical Center) or otherwise flagged as results for which equivalence to internal testing results could not be established. Whether flagged patients were being seen in the emergency department or were directly admitted to the wards, the availability of this information allowed bedside physicians to avoid unnecessary SARS-CoV-2 testing, thereby minimizing the waste of limited testing resources.

### EHR Tools and the Maintenance of PPE Supplies

Like many US health care institutions, early in the pandemic, we recognized the potential for a shortfall in the critical PPE supplies required for the care of patients with COVID-19, including N95 respirators. Providing appropriate protection to health care workers while minimizing PPE consumption made the accurate identification and flagging of PUIs essential. Our supply of N95 respirators reached a nadir in late March—less than 14 days’ worth of stock on hand overall and less than 7 days’ worth of supply for the scarcest respirator size—but subsequently recovered. Although multiple concurrent strategies, including UV reprocessing and the enhanced scrutiny of N95 respirator usage, also contributed to the successful management of this shortfall, the proper assignment of PUI statuses was a critical component in the struggle to reduce PPE use. Improvements in the national supply of N95 respirators have since reduced the acute importance of these considerations, but the strategies developed during the COVID-19 pandemic for managing limited PPE supplies will be beneficial approaches to dealing with future resource challenges.

## Results

### SARS-CoV-2 Test Ordering Metrics

The frequencies with which orders for SARS-CoV-2 tests needed to be revised due to user error or had to be repeated were used as measures for the impact of the CDS tools. The percentage of tests that were canceled and reordered due to errors in selecting the correct testing modality was 3.8% (23/608) prior to CDS implementation and 1% (262/26,643) after the implementation of CDS (Fisher exact test: *P*<.001). The percentages of patients for whom multiple tests were ordered during a 24-hour period were 0.8% (5/608) and 0.3% (76/26,643) prior to and after CDS implementation, respectively, as of October 31, 2020 (Fisher exact test: *P*=.03).

### SARS-CoV-2 Infection Frequency

If the information captured by the decision tree regarding the assignment of SARS-CoV-2 test modalities and PUI statuses accurately reflected the risk of infection, it would be expected that the incidence of positive test results would vary accordingly. Patients were classified as symptomatic or asymptomatic via the decision tree based on the presence or absence of a fever without an identified source or the presence of respiratory symptoms. Consistent with our expectations, the observed frequency of positive nasopharyngeal molecular assays for asymptomatic patients (826/24,170, 3.4%; Table 2) was significantly lower (Fisher exact test: *P*<.001) than that frequency for symptomatic patients (1421/13,074, 10.9%). Likewise, the incidence of positive test results was higher among asymptomatic patients with a history of exposure to an individual with COVID-19 (36/283, 12.7%) than among asymptomatic patients without such an exposure history (790/23,887, 3.3%; Fisher exact test: *P*<.001).

**Table 2:** SARS-CoV-2 testing volumes and results by ordering indication.

Testing indication category^a^		Testing volume^b^, N	Positive tests^b^, n (%)
**Asymptomatic patients^c^**		24,170	826 (3.4)
	Preprocedural screening^d^	12,864	428 (3.3)
	Admission screening	10,625	329 (3.1)
	Screening before behavioral health placement	398	33 (8.3)
	Admission screening of asymptomatic patients with a history of close contact with an individual with COVID-19	283	36 (12.7)
**Symptomatic patients**		13,074	1421 (10.9)
	Admission screening or hospitalized patients	5573	433 (7.8)
	Preprocedural screening^d^	298	31 (10.4)
	Outpatients with risk factors for severe illness	307	48 (15.6)
	Lower respiratory tract disease without an alternative explanation^e^	30	3 (10)
	Symptomatic patient with a history of close contact with an individual with COVID-19^e^	3	0 (0)
	Symptomatic patient without other specified criteria	6863	906 (13.2)
**Symptom status not specified**		15,341	1146 (7.5)
	Preprocedural screening^d^	6796	177 (2.6)
	Unrestricted send-out testing	5330	791 (14.8)
	Testing approved by the Division of Infectious Diseases	535	72 (13.5)
	Patient screening after health care exposure	89	2 (2.2)
	Unclassified testing	2591	104 (4)
Total testing		52,585	3393 (6.5)

^a^The testing indication categories listed summarize a larger number of actual indications displayed in the electronic health record, which were dynamically modified over the course of the pandemic.

^b^Testing data cover the period from March 13, 2020, through March 24, 2021.

^c^Patients without fevers and without respiratory symptoms were classified as asymptomatic.

^d^Includes testing before surgery and other qualifying aerosol-generating procedures.

^e^These criteria were used only briefly during the early phase of the pandemic, after which test eligibility was expanded to include symptomatic patients and tests did not need to consider these criteria.

Another group of asymptomatic patients for whom we observed a significantly increased incidence of positive SARS-CoV-2 test results included patients awaiting behavioral health placement (33/398, 8.3%; other asymptomatic patients without a history of COVID-19 exposure: 757/23,489, 3.2%; *P*<.001). The reason for this increased positivity rate is unclear, but some of these patients likely had a history of prior infection and were referred to our facilities for repeated testing before behavioral health placement to assess for viral clearance. Furthermore, the behavior patterns of these patients may have included decreased adherence to prevention measures such as mask wearing and social distancing, which placed them at an increased infection risk.

Testing for symptomatic patients when resources were the most limited was initially targeted toward those who (1) required hospitalization, (2) had comorbid conditions that increased their risk for developing a serious illness, (3) had a history of COVID-19 exposure, or (4) had a lower respiratory tract infection without another explanation. As the availability of test reagents improved, test eligibility was expanded more broadly to include symptomatic patients, and several of these more specific indications were retired. However, clinicians continued to use the decision tree to identify hospitalized patients and those with risk factors for severe illness to prioritize such patients for rapid testing. All symptomatic patients were designated as PUIs, even when the decision tree did not require more detailed information.

Symptom status was not captured for a subset of test orders (15,341/52,585, 29.2%). Many of these tests were assays that were either sent out to off-site laboratories for nonhospitalized patients or collected as screening tests several days in advance of a scheduled procedure. In the first case, symptomatic patients were instructed to isolate at home pending the result of the test. In the second case, presurgical screening results were generally available by the time patients returned for surgery. The empiric assignment of PUI statuses in the EHR at the time of testing was therefore not prioritized for these patients. Since September 2020 however, improvements in implementation resulted in the consistent capturing of symptom information for ≥80% of tested patients every month.

To manage rare or unanticipated circumstances, our testing algorithm allowed physicians in the Division of Infectious Diseases to authorize testing for patients who exhibited testing indications outside of those that were approved and implemented in the EHR. Once off-site testing became unrestricted, this approval option was used primarily for requests for locally performed tests that offered a shorter turnaround time or for patients who exhibited clinical indications that favored a specific testing platform. This approval route was needed only for 1% (535/52,585) of orders, indicating that the decision tree effectively managed a large majority of scenarios and prevented the approval activity from becoming an excessive burden on the physicians who were tasked with evaluating these nonstandard requests. The yield of positive results from such tests that were approved by infectious disease physicians was high (72/535, 13.5%), as was the frequency of positive results among unrestricted send-out tests (791/5330, 14.8%). These high rates of positive results suggest that clinicians were applying appropriate judgement to selecting patients for testing when considering these ordering options.

## Discussion

### Principal Findings

During the period following the implementation of CDS for SARS-CoV-2 test ordering, we documented improvements in the number of cancelled and reordered tests as well as decreases in the number of patients who underwent unnecessary duplicate testing. The goals of CPOE systems include submitting appropriate and efficient orders for patients [5]. Based on our data, it can be argued that this was indeed accomplished by using the decision tree for SARS-CoV-2 test ordering to help clinicians navigate the complex test eligibility criteria. However, the implementation of CPOE and CDS systems has been found to provoke strong emotions in care providers, with negative emotions being the most prevalent. In addition to contributing to the stressors that care providers already face, poorly implemented CDSs can fail if they are too cumbersome to be used as intended [18]. A successful CDS system needs to (1) provide clinicians with the best available knowledge when needed, (2) be highly adopted, (3) be effectively used, and (4) result in continuous improvements in knowledge [19].

Evaluating the effective adoption of CDS can be difficult, as care providers always have the option of selecting criteria randomly in order to complete the ordering process. When evaluating the positivity rates for the patient groups that were defined by the decision tree algorithm, we found statistically significant differences (as expected) in rates of SARS-CoV-2 test positivity between asymptomatic and symptomatic patients and between asymptomatic patients without a history of exposure to SARS-CoV-2 and asymptomatic patients with a history of such exposure. These findings suggest that clinicians appropriately used the questions in the CDS algorithm to help triage patients.

### Limitations

Our study has several limitations. First, this was an observational study and not a randomized controlled trial. Therefore, other interventions and institutional changes could have explained the decrease in order error rates. Second, the period prior to the implementation of CDS was relatively brief; during this period, a comparatively lower volume of testing was performed. Third, the decision tree was continually modified over time; new indications, such as patients awaiting behavioral health placement, were added relatively late into the pandemic. Some of the positivity rates that were observed in particular patient cohorts could have been influenced by fluctuations in the infection rate within the community.

### Conclusions

The leveraging of the EHR and implementation of the decision support algorithm resulted in the decreased incidence of order entry errors, including decreases in the percentage of cancelled and reordered SARS-CoV-2 tests and the rate of duplicate testing, and the appropriate flagging of PUIs. Collectively, these interventions optimized reagent and PPE usage and protected health care workers. The data gathered through the decision tree could be used to predict differences in the likelihood of positive test results for distinct categories of patients, suggesting that clinicians appropriately used the questions in the decision tree algorithm.

## Abbreviations

AGP: aerosol-generating procedure
CDS: clinical decision support
CPOE: computerized provider order entry
EHR: electronic health record
PPE: personal protective equipment
PUI: person under investigation
